# Validation of a set of reference genes to study response to herbicide stress in grasses

**DOI:** 10.1186/1756-0500-5-18

**Published:** 2012-01-10

**Authors:** Cécile Petit, Fanny Pernin, Jean-Marie Heydel, Christophe Délye

**Affiliations:** 1 INRA, UMR1347 Agroécologie, 17 rue Sully, 21000 Dijon, France; 2 UMR CSGA CNRS 6265 INRA 1324, Université de Bourgogne, AgroSup Dijon-uB, 21000 Dijon, France

## Abstract

**Background:**

Non-target-site based resistance to herbicides is a major threat to the chemical control of agronomically noxious weeds. This adaptive trait is endowed by differences in the expression of a number of genes in plants that are resistant or sensitive to herbicides. Quantification of the expression of such genes requires normalising qPCR data using reference genes with stable expression in the system studied as internal standards. The aim of this study was to validate reference genes in *Alopecurus myosuroides*, a grass (*Poaceae*) weed of economic and agronomic importance with no genomic resources.

**Results:**

The stability of 11 candidate reference genes was assessed in plants resistant or sensitive to herbicides subjected or not to herbicide stress using the complementary statistical methods implemented by NormFinder, BestKeeper and geNorm. Ubiquitin, beta-tubulin and glyceraldehyde-3-phosphate dehydrogenase were identified as the best reference genes. The reference gene set accuracy was confirmed by analysing the expression of the gene encoding acetyl-coenzyme A carboxylase, a major herbicide target enzyme, and of an herbicide-induced gene encoding a glutathione-S-transferase.

**Conclusions:**

This is the first study describing a set of reference genes (ubiquitin, beta-tubulin and glyceraldehyde-3-phosphate dehydrogenase) with a stable expression under herbicide stress in grasses. These genes are also candidate reference genes of choice for studies seeking to identify stress-responsive genes in grasses.

## Background

Differences in gene expression are at the root of plant adaptive response to the environment. Analysing gene expression patterns requires tools enabling sensitive, precise, and reproducible quantification of specific mRNAs. Quantitative real-time polymerase chain reaction (qPCR) is currently the technique of choice for this purpose [1]. However, technical and sample variations usually render absolute quantification of gene expression unreliable. Thus, a normalisation strategy using one, or preferably several, reference gene(s) is generally implemented in qPCR studies [1-4]. A reference gene must be constitutively and constantly expressed in all experimental conditions and samples studied [5,6]. Compared to animals, relatively few studies so far had described validated sets of reference genes in plants [7], and most of them considered species with sequenced genomes (i.e., crop or model species). Exceptions are the grass species *Lolium *sp. (e.g. [8,9]) and *Brachiaria brizantha *[10].

*Alopecurus myosuroides *(black-grass, *Poaceae*) is a grass weed of major economic importance in winter crops in Europe that is removed from crops by herbicide applications. Resistance to herbicides has evolved in numerous *A. myosuroides *field populations [11]. Resistance is mostly endowed by a range of mechanisms decreasing the amount of herbicide molecules reaching their target (non target-site-based resistance [11-13]). This type of resistance is a major threat to crop protection, because it can confer unpredictable resistance to herbicides with different modes of action [12]. As it is considered to be endowed by differential regulation of many stress-responsive genes between resistant and sensitive plants [14,15], transcriptomics-based approaches should identify genes involved. Designing a reliable qPCR assay is a prerequisite to adequately validating the gene expression data. Here, we describe the validation of a set of three reference genes in *A. myosuroides*, a non-model species with no genomic resources. These genes can reliably be used for qPCR normalisation of gene expression data in plants resistant or sensitive to herbicides before and up to at least 6 hours after herbicide application.

## Methods

### Plant material

All plants used were grown from seeds in a climatic chamber (22°C/18°C day/night; 14-h photoperiod at 450 μmol m^-2 ^s^-1^). Individual plants with a dozen tillers each were split into ten individual tillers, and every tiller was grown for 3 days in an individual pot before being used for spraying experiments [13]. All tillers from a given plant are clones, i.e., genetically identical plants at the same growth stage (3-4 leaves). To identify genes with stable expression among phenotypes (resistant *versus *sensitive to herbicides) and among experimental conditions (herbicide-treated *versus *untreated), a time-course experiment was subsequently conducted. Two clones per plant (= one sample) were collected at each of three different times (before, 2.5 hours and 6 hours after herbicide application) encompassing the most crucial period after herbicide application when expression of non-target-site-based resistance genes enables resistant plants to survive herbicide exposure [14,15]. The basal part of the clones (approximately 10 cm high and 100 mg fresh weight tissue) was cut just above the ground, immediately frozen in liquid nitrogen and stored at -80°C until RNA extraction. The basal section contains the meristematic tissues, where most of the activity of chloroplastic acetyl coenzyme A carboxylase (ACCase; EC 6.1.4.2), the target enzyme of the herbicide used, is located [14]. Two additional clones per plant were used to characterise the phenotype (i.e. resistant or sensitive). The last two clones were sprayed with water ("untreated control"). The herbicide fenoxaprop, a broadly used ACCase inhibitor, was applied using a custom-built, single-nozzle (nozzle 110-04; Albuz, France) sprayer delivering herbicide in 300 L ha^-1 ^water at 400 kPa, at a speed of 6.6 km h^-1 ^[13]. Clones from sensitive plants from a reference population [16] were used as a control for herbicide application efficacy. All clones were grown, sprayed and collected at the same time and in the same conditions. Plant survival was assessed one month after herbicide application. Dead and surviving plants were classified as sensitive and resistant, respectively.

### RNA extraction, quantification and quality assessment

Total RNA from every sample was extracted and DNA contamination removed using the RNeasy plant mini kit (Qiagen, Hilden, Germany) following the manufacturer's instructions. Nucleic acid concentration of each sample was measured twice at 260 nm using a NanoDropND-1,000 spectrophotometer (LABTECH, Luton, UK). Measurement was repeated if the two measures differed by more than 3%. Total RNA quality was assessed using the A_260_/A_280 _and A_260_/A_230 _absorption ratios. Only RNA samples with A_260_/A_280 _and A_260_/A_230 _ratios between 1.8 and 2.2 and between 2 and 2.2, respectively, were subsequently used. Total RNA integrity was checked by electrophoresis on 0.8% (w/v) agarose gels under denaturing conditions. Finally, RNA quality and genomic DNA (gDNA) contamination were assessed by capillary electrophoresis on an Experion labchip electrophoresis system (Bio-Rad, Hercules, USA). No gDNA contamination was detected. Each step from total RNA extraction to gDNA contamination assessment was performed at the same time for all samples.

Total RNA was extracted from 3 sensitive and 7 resistant plants at each of three time-course points (before, 2.5 and 6 hours after herbicide application), yielding thirty RNA samples. Following quality assessment, 19 of these samples were used for qPCR experiments (Table 1).

**Table 1:** RNA samples used for assessment of the stability of the candidate reference genes

Plant phenotype	Herbicide application			Total
	**BEFORE treatment**	**AFTER treatment**		

		**+2.5H**	**+6H**	

SENSITIVE	1	2	2	5

RESISTANT	4	5	5	14

Total	5	7	7	19

Samples were assigned to five subsets according to plant phenotype or to the time after herbicide application. Subsets containing samples collected at the same time (BEFORE, + 2.5H or + 6H) enabled to assess gene stability between phenotypes (i.e. phenotype effect). Subsets containing samples from plants with a same phenotype (SENSITIVE or RESISTANT) enabled to assess the effect of the time after herbicide application on gene stability.

### cDNA synthesis

cDNA synthesis was performed in duplicate for every RNA sample that passed the quality controls. Reverse-transcription (RT) reactions were performed simultaneously for all samples. cDNA was synthesized from 5 μg of total RNA using the two-step RT-PCR protocol of the Masterscript RT-PCR System (5 PRIME, Hamburg, Germany). Both the oligo(dT)_18 _primer (0.5 μg) and the random primers (50 ng) were used with 1 μL reverse-transcriptase in a 20 μL-reaction volume following the manufacturer's instructions. The two-step protocol reduces unwanted primer dimer formation [17], which must be avoided when using SYBR Green as the qPCR quantification dye. Reactions were immediately stored at -20°C until further use. To detect gDNA contamination, all cDNA samples and a sample of *A. myosuroides *gDNA were simultaneously used in PCRs to amplify a fragment of *ACCase *[genbank: AM408429] using intron-spanning primers ACVII8 (5'- AGGACACGCAGAGGAACCTCTTTCATTTAC) and ACVII8R (5'- CAACTCTCCAGCTACCACTGGCAGG). Amplicon sizes (340 bp for cDNA and 426 bp for gDNA) were compared on 2.5% (w/v) agarose gels. No gDNA contamination was detected.

### Primer design

Eleven genes commonly reported as stable reference genes in grasses and/or in studies investigating plant response to stress were selected. They are involved in biosynthesis pathways [glyceraldehyde-3-phosphate dehydrogenase (*GAPDH*), sucrose phosphate synthase (*SPS*) and ribulose biphosphate carboxylase (*RUBISCO*)], cytoskeletal structure [beta-tubulin (*TUB*) and actin (*ACT*)], protein metabolism [elongation factor 1α (*EF1*), ubiquitin (*UBQ*) and cyclophilin (*CYC*)], and ribosomal structure [nucleus-encoded 18S rRNA (18*S*) and 25S rRNA (25*S*), and mitochondrial-DNA-encoded 26S rRNA (26*S*)].

Hardly any genomic data is available for *A. myosuroides*. Primers for reference genes (Table 2) were therefore designed based on conserved regions in the homologous genes in other grasses (rice, barley, wheat, maize, *Lolium sp*. and *Brachypodium distachyon*; Additional file 1: Table S1). Primers were designed using Primer3 [18], using a primer length = 23 ± 3 bp, melting temperature (Tm) = 60°C ± 3°C, a guanine-cytosine content around 50%, and an expected amplicon size of 150 to 250 bp. To detect gDNA contamination, the primers targeting *TUB *or *GAPDH *were designed to amplify an intron-containing amplicon. Primers were synthesized by MWG Biotech (Germany, Ebersberg).

**Table 2:** Candidate reference genes tested and primer sequences

Reference gene^1 ^(accession number)	Primer sequences (5'-3')^2^	Amplicon lenght (bp)	Tm (°C)	Manual threshold^3^	PCR efficiency (%)	Regression coefficient (R^2^)	Average Cq value
*CYC*	F: AGCTTTGAAGTTGGCAGTAG						
							
	R: GATCGCGTATTCATGGACTTTAG	Discarded (aspecific amplification)					
						
*SPS*	F: CATTGCAAGAACTATTTGTCACG						
							
	R: GCAGAGATCAAATGGTTCAAATC						

*ACT*	F: TGTGCTTGACTCTGGTGATG	220	58				
							
	R: TTCATAATCAAGGGCAACGTAAGC			Discarded (aspecific amplification)			
				
*RUBISCO*	F: CATTATCAAGAAGGGCAAGATGTG	169	60				
							
	R: TGTTGTACATCCCTGGAAGTTG						

*GAPDH*	F: GTATTGTTGAGGGACTGATGACC	182	57	0.047	92	0.999	23.31
							
(JN599100)	R: AGTAAGCTTGCCATTGAACTCAG						

*TUB*	F: TACTGTGGTTGAGCCATACAATG	162	60	0.069	98	0.993	23.87
							
(JN599101)	R: GCAGAGATCAAATGGTTCAAATC						

*EF1*	F: CAAGTACTACTGCACCGTCATTG	199	57	0.025	89	0.984	25.10
							
(JN599095)	R: GATCATCTGCTTCACTCCAAGAG						

*UBQ*	F: GCAAGAAGAAGACCTACACCAAG	225	60	0.054	100	0.991	19.00
							
(JN599096)	R: CCTTCTGGTTGTAGACGTAGGTG						

*18S*	F: GTCCAGACATAGGAAGGATTGAC	245	63	0.052	106	0.995	15.08
							
(JN599097)	R: GAACATCTAAGGGCATCACAGAC						

*25S*	F: GCATGAATGGATTAACGAGATTC	165	63	0.098	95	0.998	17.00
							
(JN599099)	R: GGCTCCCACTTATCCTACAC						

*26S*	F: GATAGCGTACAAGTACCGTGAGG	238	63	0.102	94	0.993	20.45
							
(JN599098)	R: GTTTCGGGTCAAATAGGAAGAAC						

^1^CYC cyclophilin, *SPS *sucrose phosphate synthase, *ACT *actin, *RUBISCO *ribulose biphosphate carboxylase, *GAPDH *glyceraldehyde-3-phosphate dehydrogenase, *TUB *beta-tubulin, *EF1 *elongation factor 1α, *UBQ *ubiquitin, *18S *18S ribosomal RNA (nuclear gene), *25S *25S ribosomal RNA (nuclear gene), *26S *26S ribosomal RNA (mitochondrial gene)

^2^F: forward primer and R: reverse primer

^3^The threshold for fluorescence detection was set using the logarithmic amplification plot so that it is above the background fluorescence, below the linear region and at the beginning of the region of exponential amplification (i.e. the linear portion of the plot)

### PCR

Specific amplification from cDNA and gDNA was checked by PCR followed by electrophoresis on 2% (w/v) agarose gels, and the annealing temperature optimised where necessary. PCR mixes were as described [16]. Amplicons were sequenced to confirm amplification of the targeted gene *in A. myosuroides *(accession numbers are in Table 2).

### QPCR

qPCR was performed in fast optical 0.1 ml, 96-well reaction plates (MicroAmp™, Applied Biosystems, Cheshire, UK) using the ABI PRISM 7,900 HT Sequence Detection System (Applied Biosystems, Foster City, USA). The reaction volume (20 μl) contained 2 μl of a cDNA RT mix diluted 125-fold, 10 μl of ABsolute QPCR SYBR Green ROX mix (ThermoScientific, Epsom, UK) and 0.5 μM of each gene-specific primer. Polymerase activation (95°C for 15 min) was followed by 40 quantification cycles [95°C for 15 s, Tm (Table 2) for 30s and 72°C for 30s]. After 40 cycles, a melting-curve analysis (68°C to 95°C, one fluorescence read every 0.3°C) was performed to check the specificity of the amplifications. Amplicon sizes were checked on 3% (w/v) agarose gels. PCRs with each primer pair were also performed on three samples lacking cDNA template (negative controls).

To assess the amplification efficiency of each candidate gene, identical volumes of all cDNA samples (2 RT reactions per RNA sample) were pooled. The pool was diluted and used to generate five-point standard curves based on a five-fold dilution series (1:5-1:3125). This was performed in duplicate. Each duplicate series was amplified in two independent qPCR runs. Amplification efficiency (E) was computed as: E = 10^(-1/a) ^-1 [19], where a is the slope of the linear regression model (y = a log(x) + b) fitted over log-transformed data of the input cDNA concentration (y) plotted against quantification cycle (Cq) values (x). The four E-values obtained from the dilution series were averaged for each primer pair. E-values for different target genes were considered comparable when included in the range of 100 ± 10% (standard curve slope of -3. 3 ± 0.33) [20].

To assess gene stability, all cDNA samples that passed quality assessment were diluted 125-fold. For each gene, every diluted sample was amplified twice in two independent qPCR runs. As two independent RTs were performed per RNA sample, this yielded four technical replicates per RNA sample.

Data were analysed using SDS 2.3 (Applied Biosystems, Foster City, USA). To generate a baseline-subtracted plot of the logarithmic increase in fluorescence signal (DRn) against cycle number, baseline data were collected between qPCR cycles 3 and 15, so that the amplification curve growth begins at a cycle number greater than the stop baseline cycle. The threshold for fluorescence detection was set using the logarithmic amplification plot so that it is above the background fluorescence, below the linear region and at the beginning of the region of exponential amplification.

### Data analysis

To evaluate the stability of candidate reference genes expressed as Cq values, we used BestKeeper [21], geNorm v. 3.5 [4] and NormFinder [22]. NormFinder and geNorm require the transformation of Cq values by the 2^-ΔΔCq ^method [20], using the lowest Cq as a calibrator. BestKeeper first computes the variation in Cq values and its standard deviation (SD) for each gene. Genes with SD > 1 are considered unstable [21]. The remaining genes are ranked based on pairwise correlations between the Cq values of each gene and the geometric mean of the Cq values of all genes (BestKeeper Index). Candidate genes showing the strongest correlation with the BestKeeper Index are considered the most suitable [21]. NormFinder ranks the candidate genes after their Stability Values (SV) based on the variations of their respective transformed-Cq values within and among groups [22]. Reference genes with the lowest SVs are top ranked, and considered the most suitable reference genes. NormFinder subsequently computes the SV of the combination of the two most stable genes. geNorm computes all possible average pairwise variation between the candidate gene transformed-Cq values, and provides a measure of the expression stability (M) of each gene. An M value below 1.5 identifies stable reference genes [4]. geNorm then performs stepwise exclusion of the gene with the highest M-value (least stably expressed gene) and recalculates M values for the remaining genes. This iterative process enables to rank candidate genes based on their stability of expression. As a single reference gene may not allow adequate normalisation, geNorm computes the optimal number of reference genes required for accurate normalisation by calculating pairwise variations V_n/n+1 _between consecutively ranked normalisation factors NF_n _and NF_n+1_, where n and n+1 are the number of genes considered, and NF_i _are the geometric means of the i best candidate reference gene transformed Cq values. A pairwise variation of 0.15 is suggested as a cut-off value below which the inclusion of an additional reference gene is not required for reliable normalisation [4].

### Application: Expression level of *ACCase *and *GSTL *in *A Myosuroides*

*UBQ, GADPH *and *TUB *were used to normalise the expression data of two genes. Expression data were generated using all cDNA samples that passed quality assessment. *ACCase *that encodes the target of the herbicide used in this study is expected to be stably expressed in herbicide-resistant compared to herbicide-sensitive plants [14,23]. *GSTL *that encodes a lambda-class glutathione-S-transferase had been shown to be up-regulated in herbicide-resistant plants compared to sensitive plants [24]. Amplification of a 175 bp fragment of *ACCase *[genbank: AM408429] was performed using primers ACVII 27 (5'-CACAAGATGCAGCTAGATAGTGGCG) and ACVII34R (5'-TTCCAACAGTTCGTCCAGTAACGAATG) with a Tm of 60°C. Amplification of a 156 bp fragment *of a GSTL *[genbank: FN394979, FN394980, FN394981] was performed using primers VGSTL-3 (5'-GGTTCAGATATACTATTCTCACC) and VGSTL-3R (5'-CTTGAGATGCCTCTTGGCAAC) with a Tm of 60°C. qPCR results were analysed using REST 2009 ver. 2.0.13 [25] that compares the expression level of a target gene in a 'sample' group using a 'control' group as a reference, taking into account the respective amplification efficiencies of each reference gene and target gene computed as described in the 'qPCR' section. Gene expression data analysed consisted into average Cq values computed for two replicates (two independent qPCR runs, each performed from one of two independent RT reactions). REST 2009 implements the 2^-ΔΔCq ^method to the transformation of Cq values [20]. *ACCase *and *GSTL *expression was compared in sensitive plants ('control') and in resistant plants ('sample') before, 2.5 hours and 6 hours after herbicide application, using a pairwise fixed reallocation randomisation test (2,000 iterations).

## Results

### Specificity and efficiency of amplification

PCRs using primers targeting *CYC *and *SPS were *aspecific, and melting curve profiles revealed primer dimer formation or aspecific amplification for primers targeting *ACT *or *RUBISCO *(Additional file 2: Figure S1A and B). Other sets of primers designed and tested for these four genes did not increase the specificity of amplification (not shown). These four genes were thus not further considered (Table 2).

**Figure 1:**
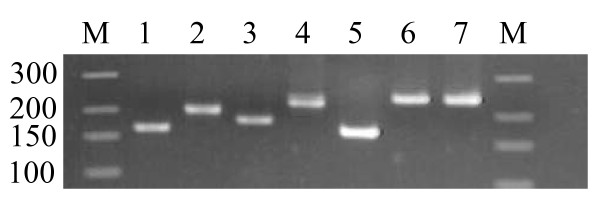
**Agarose gel (3%) electrophoresis showing amplicon size for seven candidate reference genes**. These genes all passed the specificity and efficiency assessment steps. 1, TUB; 2, EF1; 3, GAPDH; 4, UBQ; 5, 25S; 6, 26S; 7, 18S. M, DNA ladder (the size of the DNA ladder fragments is given on the left in base pairs).

The single-peak melting curves obtained for the seven remaining candidate genes confirmed the absence of primer dimers or non-specific products (Additional file 2: Figure S1 C-I). The no-template controls (NTCs) yielded Cq values comprised between 31 and 35 cycles. The corresponding melting curves showed a small peak located before the position of the amplicon peak observed in the template samples. As no amplicon peak was detected in the NTCs, the positive Cq values observed were attributed to primer dimer formation, and were ignored. Specific amplification of the expected amplicons was confirmed by agarose gel electrophoresis (Figure 1).

qPCR efficiency and correlation coefficients computed from the five-fold dilution series are given in Table 2. The amplification efficiency of *EF1 *(89%) was just below the 90% cut-off value. As amplification was specific for *EF1*, it was included in the subsequent analyses. All six remaining genes fulfilled our criteria for specific and effective amplification (Table 2).

### Stability of candidate reference genes

For every gene, qPCR data consisting into the mean Cq value computed from four technical replicates was generated from each of the 19 RNA samples that passed quality control (Table 1). The technical replicates consisted into two independent qPCR runs performed on each of two independent RT reactions per RNA sample. Expression data was subdivided in five subsets according to the phenotype or to the time after herbicide application (Table 1).

#### BestKeeper analyses

Gene stability was assessed considering all samples as a single set where all possible combinations of the tested effects (time after herbicide application and phenotype) were present. All genes except *EF1 *were found suitable for normalisation (SD < 1, Table 3; Additional file 1: Table S2). Among the remaining 6 genes, the highest r values were observed for *TUB*, *26S*, *GAPDH *and *UBQ*. *TUB *was ranked the second most stable gene because of its SD value (1.07). *26S *that had the lowest SD value and the second highest r value was ranked the most stable gene (Table 3; Additional file 1: Table S2).

**Table 3:** Ranking of the candidate reference genes according to their stability value using BestKeeper and NormFinder

	**BestKeeper**					**NormFinder**				
	
**Reference genes^a^**	**All samples**				**All samples**		**Herbicide effect**		**Phenotype effect**	
		
							**BEFORE/+2.5H+6H**		**SENSITIVE/RESISTANT**	
		
	**SD^b^**	**r ^c^**	**Ranking order**		**SV**^**d**^	**Ranking order**	**SV**	**Ranking order**	**SV**	**Ranking order**
		
*26S*	0.46	0.80*	**1**		0.335	**1**	0.148	**1**	0.132	**1**
		
***TUB***	1.07	0.88*	**2**		0.488	**2**	0.204	**4**	0.156	**2**
		
***GAPDH***	0.93	0.70*	**3**		0.494	**3**	0.188	**3**	0.242	**4**
		
***UBQ***	0.81	0.67	**4**		0.515	**4**	0.175	**2**	0.254	**5**
		
*25S*	0.50	0.53	**5**		0.669	**5**	0.258	**5**	0.279	**6**
		
*18S*	0.96	0.42	**6**		1.039	**7**	0.378	**7**	0.370	**7**
		
*EF1*	1.45	0.85*	**7**		0.813	**6**	0.332	**6**	0.231	**3**

^a^The three reference genes selected are in bold

^b^Standard deviation of Cq values. SD values higher than the cut-off value (1.00) are underlined

^c^Pearson coefficient of correlation

^d^Stability value

*: associated p value <0.001

#### Norm Finder analyses

To assess the effect of the plant phenotype and of the time after herbicide application on gene stability, we analysed three different data sets comprising all samples 1- without subset assignment; 2- with every sample assigned to one of the subsets BEFORE, + 2.5H or + 6H and 3- with every sample assigned to one of the two subsets SENSITIVE or RESISTANT. *26S*, *TUB*, *GAPDH *and *UBQ *were the most stable genes overall and considering the time after herbicide application (Table 3). *26S*, *TUB*, *GAPDH *and *EF1 *were the most stable genes between sensitive and resistant plants (i.e. phenotype effect) (Table 3).

When the samples were not assigned to a subset or assigned to subsets SENSITIVE or RESISTANT, the combination of the two most stable genes was *26S *and *TUB *(Table 3). This combination had a SV value (0.092) lower than that of the most stable gene (*26S*, SV = 0.132; Table 3). When the samples were assigned to the subsets BEFORE, + 2.5H and + 6 H, the most stable combination of two genes was *26S *and *UBQ*, with a SV value (0.119) lower than that of the most stable gene (*26S*, SV = 0.148; Table 3).

Overall, *26S*, *TUB*, *UBQ *and *GAPDH *were identified as the most stable genes.

#### geNorm analyses

The subsets BEFORE and SENSITIVE (five replicates per subset, Table 1) did not contain enough biological replicates to allow reliable analysis. Thus, we first used the whole data set to assess gene stability when the two effects (i.e. plant phenotype and time after herbicide application) are combined. We subsequently analysed each of the subsets + 2.5H and + 6H independently to assess phenotype effect, and the subset RESISTANT to assess the effect of time after herbicide application.

**Figure 2:**
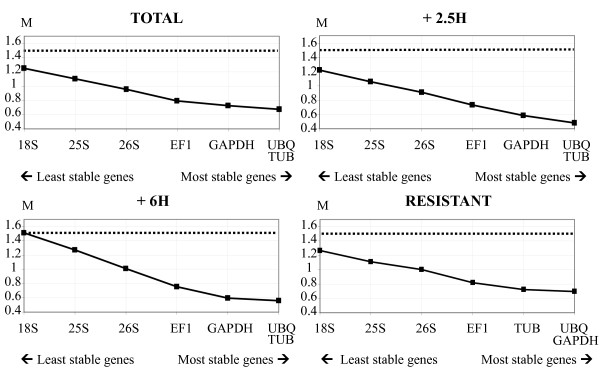
**Average expression stability values (M) of seven candidate reference genes computed using geNorm**. Ranking was performed for all RNA samples (TOTAL), and for the subsets +2.5H, +6H and RESISTANT. M-values of the remaining genes at each step during stepwise exclusion of the least stable gene are shown. The genes are ranked according to increasing expression stability (decreasing M-values) starting from the least stable gene on the left. The two most stable genes are on the right. The horizontal dotted line indicates the cutoff M-value (1.5).

In all analyses, all genes showed M values below the default cutoff value (1.5) (Figure 2). Overall, *UBQ*, *TUB *and *GAPDH *were the three most stable genes, *UBQ *and *TUB *being the most stable among all samples and between contrasted phenotypes, and *UBQ *and *GAPDH *being the most stable when considering the effect of the time after herbicide application (Figure 2).

**Figure 3:**
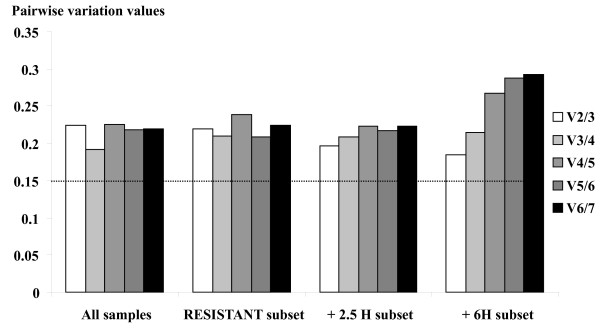
**Determination of the optimal number of reference genes for accurate normalisation. geNorm calculates pairwise variations (V**_**n/n+1**_**) between consecutively ranked normalisation factors NF**_**n **_**and NF**_**n+1 **_**(NF**_**i **_**is the geometric mean of the expression values for the i first-ranked candidate reference genes)**. Each pairwise variation value is compared to the cutoff value (0.15, *horizontal dotted line*). For example, V_2/3 _corresponds to the pairwise variation between the normalisation factors of the two first-ranked (2) and the three first-ranked (3) reference genes.

The optimal number of reference genes required for accurate normalisation was computed. All values obtained for the pairwise variation between consecutive normalisation factors (V_i/j _values, Figure 3) were higher than the proposed 0.15 cutoff threshold [4]. As recommended in this case [4], we considered the change in the V_i/j _values when including additional reference genes, and we used the lowest V_i/j _value to determine the number of reference genes adequate for normalisation. Regarding the phenotype effect (assessed from subsets + 2.5 H and + 6H; Figure 3), the inclusion of the third most stably expressed gene yielded the lowest variation of the normalisation factor (V_2/3_), indicating that using three genes (*UBQ*, *TUB *and *GAPDH*) was adequate for normalisation. Overall (TOTAL subset) and when considering the effect of the time after herbicide application (assessed from subset RESISTANT; Figure 3), the inclusion of the fourth most stably expressed gene yielded the lowest variation of the normalisation factor (V_3/4_), indicating that using four genes (*UBQ*, *TUB*, *GAPDH *and *EF1*) was adequate for normalisation.

### Expression level of *ACCase *and *GSTL *in *A. Myosuroides*

**Figure 4:**
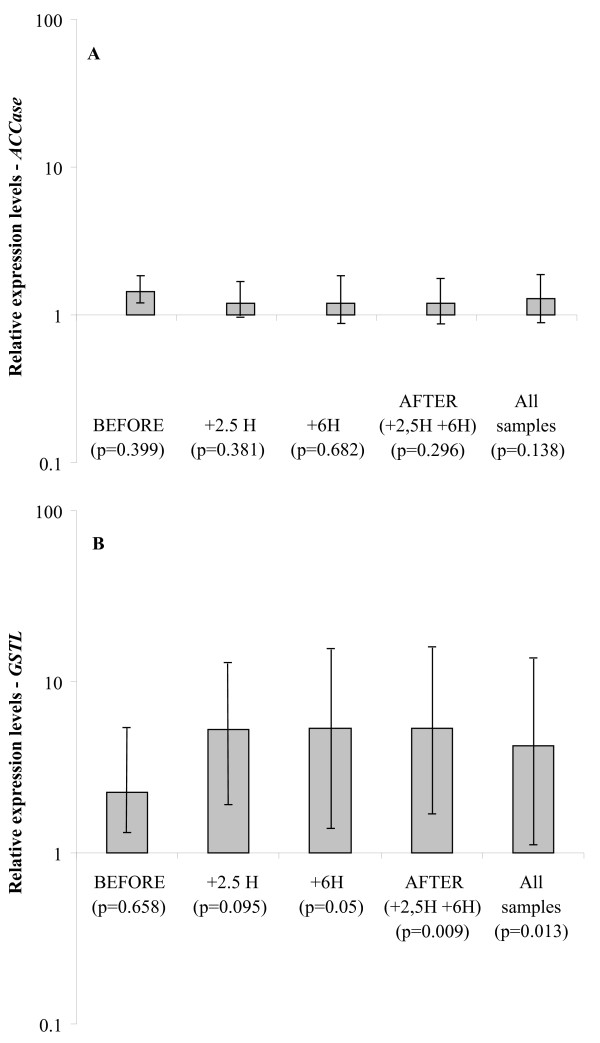
**Relative expression levels of ACCase (**a**) and GSTL (**b**) in resistant *versus *sensitive plants**. *UBQ*, *TUB *and *GADPH *were used for normalisation. Each measurement consists of two repetitions. BEFORE, before herbicide application; +2.5H and +6H, 2.5 and 6 h after herbicide application, respectively; AFTER, after herbicide application (2.5H and +6H); TOTAL, all samples. p values for differences in gene expression are given in brackets.

The expression of *ACCase *and *GSTL *was normalised using *UBQ*, *TUB *and *GAPDH*, and compared in herbicide-resistant or sensitive plants overall and in each of the subsets BEFORE, +2.5H, +6H and AFTER (Table 1). *ACCase *showed an average Cq value of 25.88 (PCR efficiency = 105%; R^2 ^= 0.997; manual threshold = 0.235). No significant differences in the expression of *ACCase *were observed between resistant and sensitive plants for all conditions tested (Figure 4a). *GSTL *showed an average Cq value of 30.11 (PCR efficiency = 109%; R^2 ^= 0.980; manual threshold = 0.249). *GSTL *was found to be 4.3-fold up-regulated in resistant plants compared to sensitive ones when considering all samples (Figure 4b). *GSTL *was 5.4-fold and 5.3-fold up-regulated when considering the two subsets + 6H and AFTER (+2.5H and +6H), respectively (Figure 4b). No significant differences in the expression of *GSTL *were observed when comparing resistant and sensitive plants before and 2.5 hours after treatment (Figure 4b).

## Discussion

The aim of this study was to identify suitable reference genes for the normalisation of gene expression data in *A. myosuroides *plants with contrasted phenotypes (sensitive or resistant) that had been submitted or not to herbicide stress. As no single method is generally accepted to test for the stability of candidate reference genes, we used the algorithms implemented by three different programs.

BestKeeper is a useful first approach generating descriptive statistics and coefficients of correlation. geNorm is considered one of the best methods to determine the most stable genes in plant studies (e.g. [7,26,27]). geNorm also indicates the optimal number of genes required for normalisation in a given experimental dataset [4]. However, it has a tendency to assign close ranks to co-regulated genes, which expression ratios show less pairwise variation than those of independently regulated genes [4]. Therefore, we also used NormFinder [22] that ranks candidate reference genes according to the variation of their expression within and among experimental modalities, and is less sensitive to possible bias arising from co-regulation [2].

The results yielded by the three programs were very similar (Table 3, Figure 2), and indicated that the most stable genes in our system were *UBQ*, *TUB *and *GAPDH*, which were always ranked among the four most stable genes. geNorm indicated that four genes would be most adequate for normalisation, also including *EF1*. However, because *EF1 *was ranked among the two least stable genes by the two other programs, we did not include it in the reference gene set. Furthermore, in most cases, using the three 'best' reference genes is a valid normalisation strategy [4].

*26S *was top-ranked by NormFinder and BestKeeper, but ranked fifth by geNorm (Figure 2). This could be explained by the sensitivity of geNorm to the co-regulation of the genes tested. It is indeed reasonable to consider that *26S*, a ribosomal RNA gene, may be co-regulated with *18S *and *25S*, ribosomal RNA genes that were consistently ranked among the least stable genes (Table 3). The high abundance of ribosomal RNAs compared with mRNAs makes it difficult to accurately subtract the baseline value in qPCR data analysis [4]. Furthermore, ribosomal RNA seems less affected than mRNA by partial degradation, which may introduce bias in normalisation [28]. Considering all these points, and because *UBQ*, *TUB *and *GAPDH *used together enabled reliable normalisation (Figure 4a-b), we did not include *26S *in the reference gene set. The combination of *UBQ*, *TUB *and *GAPDH *was thus selected to normalise gene expression data in herbicide resistance studies in *A. myosuroides*.

To check the accuracy of this set of reference genes, we investigated the expression profiles of two genes of interest in resistant and sensitive plants. Differential regulation of *ACCase *had never been shown to confer resistance to ACCase inhibitors [14,23]. Thus, *ACCase *is expected to have a similar expression level in plants sensitive or resistant to the ACCase inhibitor fenoxaprop, which was observed (Figure 4a). In contrast, *GSTL *was expected to be up-regulated after herbicide application in resistant plants compared to sensitive ones [24], which was observed considering all samples. The expression level of *GSTL *was not significantly different between resistant and sensitive plants before and 2.5 hours after treatment, but was up-regulated in resistant plants six hours after treatment (Figure 4b). This suggests an herbicide-induced up-regulation of *GSTL *in resistant plants compared to sensitive plants, which is consistent with a previous study [24]. These results thus confirm that *UBQ*, *TUB *and *GAPDH *can reliably be used as a set of reference genes to normalise qPCR data in studies on herbicide action in *A. myosuroides*.

Herbicides are an abiotic stress. Previous works identified reference genes with stable expression under different abiotic or biotic stresses in a range of plant species. *UBQ *had been shown to be stable under abiotic stresses in grasses [8,26]. *UBQ *was also stable in the broadleaf *Arabidopsis thaliana *under herbicide stress [29]. *TUB *was among the most stable genes reported in several cereal (grasses) species under different stresses [30]. *GAPDH *showed a stable expression during stress in grasses [30-32], although some stresses induced a high variability in its expression in wheat [33].

*EF1 *and *18S *were among the least stably expressed genes in our study. They are among the most commonly used reference genes in plant studies, with a stable expression reported in grasses under different stresses in several studies (e.g. [8,26,31,32]). However, other studies reported an unstable expression of these genes in grasses under biotic stress (e.g., [30,32]). This was also observed for herbicide stress in our work (Table 3). Our results and the literature thus clearly confirm the need for a thorough validation of the stability of expression of candidate reference genes in the system considered prior to any gene expression study.

## Conclusions

This is the first study describing a set of reference genes in a non-model grass plant species that is also a weed of economic and agronomic importance. To the best of our knowledge, this is also the first study conducted on a non-model plant species demonstrating the stability of expression of a set of reference genes under herbicide stress and in contrasted herbicide-related phenotypes (sensitive and resistant).

In grass weeds, herbicide resistance is mostly endowed by non-target-site-based mechanisms [11,12,23], an adaptive trait under polygenic control endowed by constitutive and/or induced differential expression of numerous genes in resistant *versus *sensitive plants [15]. Hardly any such genes have been identified so far in a weed species [14,23]. The reference gene set validated for *A. myosuroides *will be of immense help to identify genes involved in non-target-site-based resistance. Given that the expression of these reference genes seems stable in grasses, they are clearly candidate reference genes of choice for studies seeking to identify herbicide-responsive genes in other grasses with no associated genomic resources (i.e., most grass weeds), and also very likely to identify stress-responsive genes in this taxon.

## Competing interests

The authors declare that they have no competing interests.

## Authors' contributions

All authors participated in the design of the study. CP and FP carried out the experiments and performed the analyses. CP drafted the manuscript. JMH participated to the analyses of the study and helped to draft the manuscript. CD participated in the coordination of the study and critically revised the manuscript. All authors contributed to, read and approved the final manuscript.

## Supplementary Material

Additional file 1 **Table S1**. Accession numbers of the sequences used for primer design. **Table S2**. Descriptive statistics of reference gene expression in black-grass based on the BestKeeper approach. Click here for file

Additional file 2 **Figure S1**. Primer specificity test. - melting curve generated for RUBISCO. Melting curves generated for *RUBISCO *(A), *ACT (B), UBQ *(C), *EF1 *(D), *GAPDH *(E), *TUB *(F), *25S *(G), *26S *(H) and *18S *(I). Click here for file
