# Supplementary material for: Validation of a set of reference genes to study response to herbicide stress in grasses
Source: BMC Res Notes. 2012 Jan 10;5:18. doi: 10.1186/1756-0500-5-18 (PMC3292489; doi:10.1186/1756-0500-5-18)
Supplement: Additional file 1 — Table S1. Accession numbers of the sequences used for primer design. Table S2. Descriptive statistics of reference gene expression in black-grass based on the BestKeeper approach. [file 1756-0500-5-18-S1.DOC]

**Table S1 -** Accession numbers of the sequences used for primer design

| Gene | Code | Grass species | | | | | |
| --- | --- | --- | --- | --- | --- | --- | --- |
|  |  | *Brachypodium distachyon*^1^ | Rice | Wheat | Maize | Barley | *Lolium sp.* |
| glyceraldehyde-3-phosphate dehydrogenase | *GAPDH* | lcl|super_3:7976927-7986927 | AK064960 | AF251217 | U45856 | M36650 |  |
| sucrose phosphate synthase | *SPS* |  | D45890 |  |  |  |  |
| ribulose biphosphate carboxylase | *RUBISCO* |  | NM_001075087 |  |  |  |  |
| beta-tubulin | *TUB* | lcl|super_4:23389534-23399534 | D30716 | U76745 | NM_001112218 | AM502853 |  |
| actin | *ACT* | lcl|super_2:10053641-10063641 | AK100267 | AB181991 | AY107106 | AY145451 |  |
| elongation factor 1α | *EF1* | lcl|super_0:34726812-34736812 | AK061464 | AF479046 | D45408 | Z50789 | EU328532 |
| ubiquitin | *UBQ* | lcl|super_4:5791934-5801934 | AK061988 | AY290730 | BT017442 | M60175 |  |
| cyclophilin | *CYC* |  | NM_001068001 |  |  |  |  |
| 18S rRNA (nucleus) | *18S* | lcl|super_226 | AK059783 |  |  |  |  |
| 25S rRNA (nucleus) | *25S* | lcl|super_213 | AK119809 |  |  |  | EF463062 |
| 26S rRNA (mitochondria) | *26S* | lcl|super_17:453187-463187 |  | M37274 |  |  |  |

^1^ Accession numbers from the *Brachypodium* *distachyon* database (<http://www.brachypodium.org/>)

**Table S2 - Descriptive statistics of reference gene expression in black-grass based on the BestKeeper approach**

| **Rank** | **1** | **2** | **3** | **4** | **5** | **6** | **7** |
| --- | --- | --- | --- | --- | --- | --- | --- |
|  | ***26S*** | ***TUB*** | ***GAPDH*** | ***UBQ*** | ***25S*** | ***18S*** | ***EF1*** |
| n | 19 | 19 | 19 | 19 | 19 | 19 | 19 |
| GM [CP] | 20.44 | 23.83 | 23.29 | 18.97 | 16.98 | 15.02 | 25.04 |
| AM [CP] | 20.45 | 23.86 | 23.31 | 19.00 | 17.00 | 15.08 | 25.10 |
| min [CP] | 19.18 | 22.05 | 21.58 | 17.38 | 15.72 | 12.66 | 22.90 |
| max [CP] | 21.47 | 27.08 | 25.15 | 21.78 | 19.77 | 18.41 | 28.72 |
| SD [± CP] | 0.46 | 1.07 | 0.93 | 0.81 | 0.50 | 0.96 | 1.45 |
| CV [% CP] | 2.24 | 4.48 | 4.01 | 4.26 | 2.94 | 6.40 | 5.78 |
| coeff. of corr. [r] | 0.80 | 0.88 | 0.70 | 0.67 | 0.53 | 0.42 | 0.85 |
| p value | <0.001 | <0.001 | <0.001 | 0.002 | 0.019 | 0.072 | <0.001 |

*Note*: n, number of *A. myosuroides* cDNA samples; GM, geometric mean of Cq value; AM, arithmetic mean of Cq value; min and max, extreme values of Cq; SD [± CP] and, standard deviation of Cq value; CV [% CP], coefficient of variance expressed as percentage of Cq value; r, Pearson coefficient of correlation; p value, p value associated with the Pearson coefficient of correlation.
