# Supplementary figures and images for: Validation of a set of reference genes to study response to herbicide stress in grasses
Source: BMC Res Notes. 2012 Jan 10;5:18. doi: 10.1186/1756-0500-5-18 (PMC3292489; doi:10.1186/1756-0500-5-18)

## Slide 1
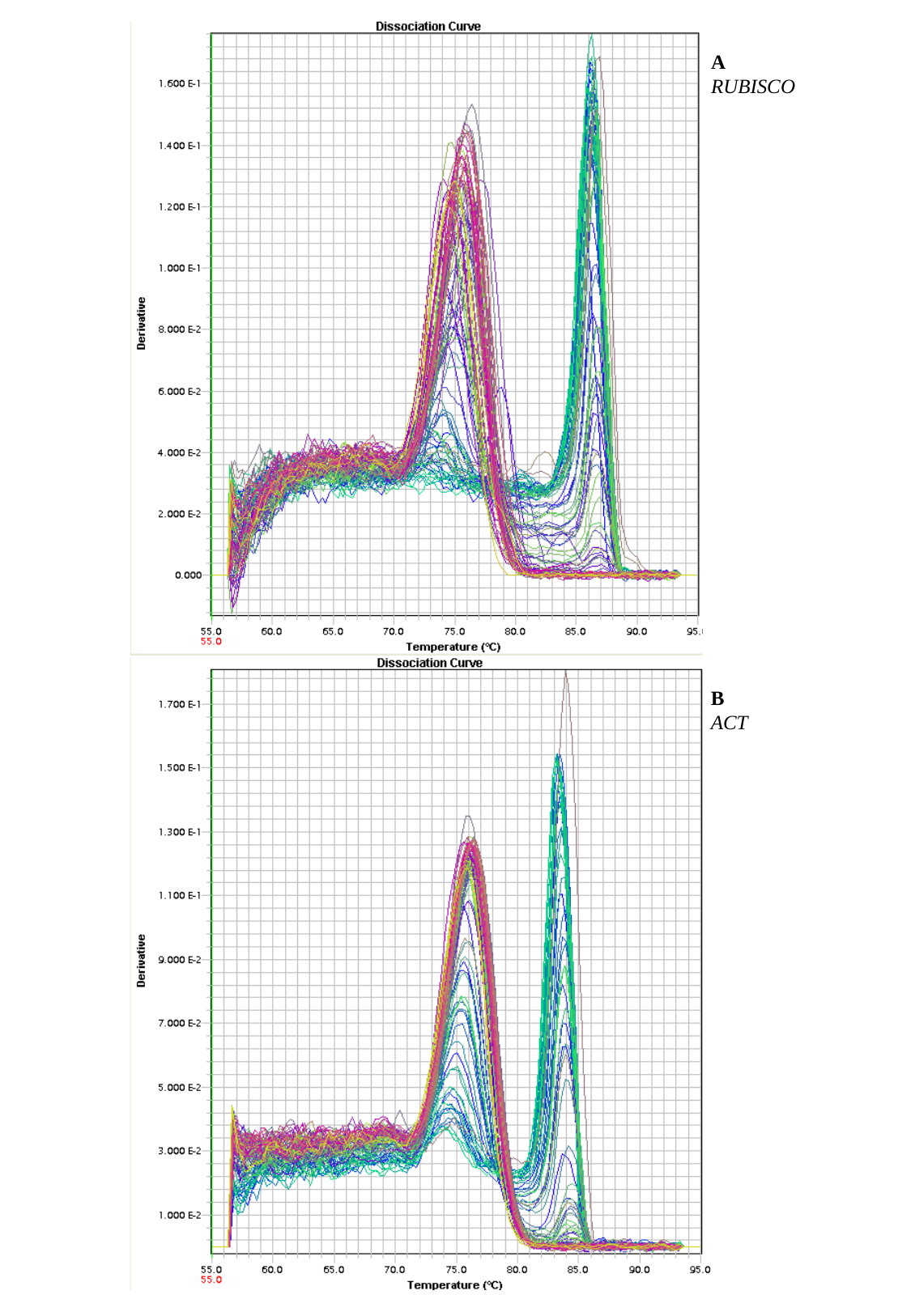

A
RUBISCO
B
ACT

## Slide 2
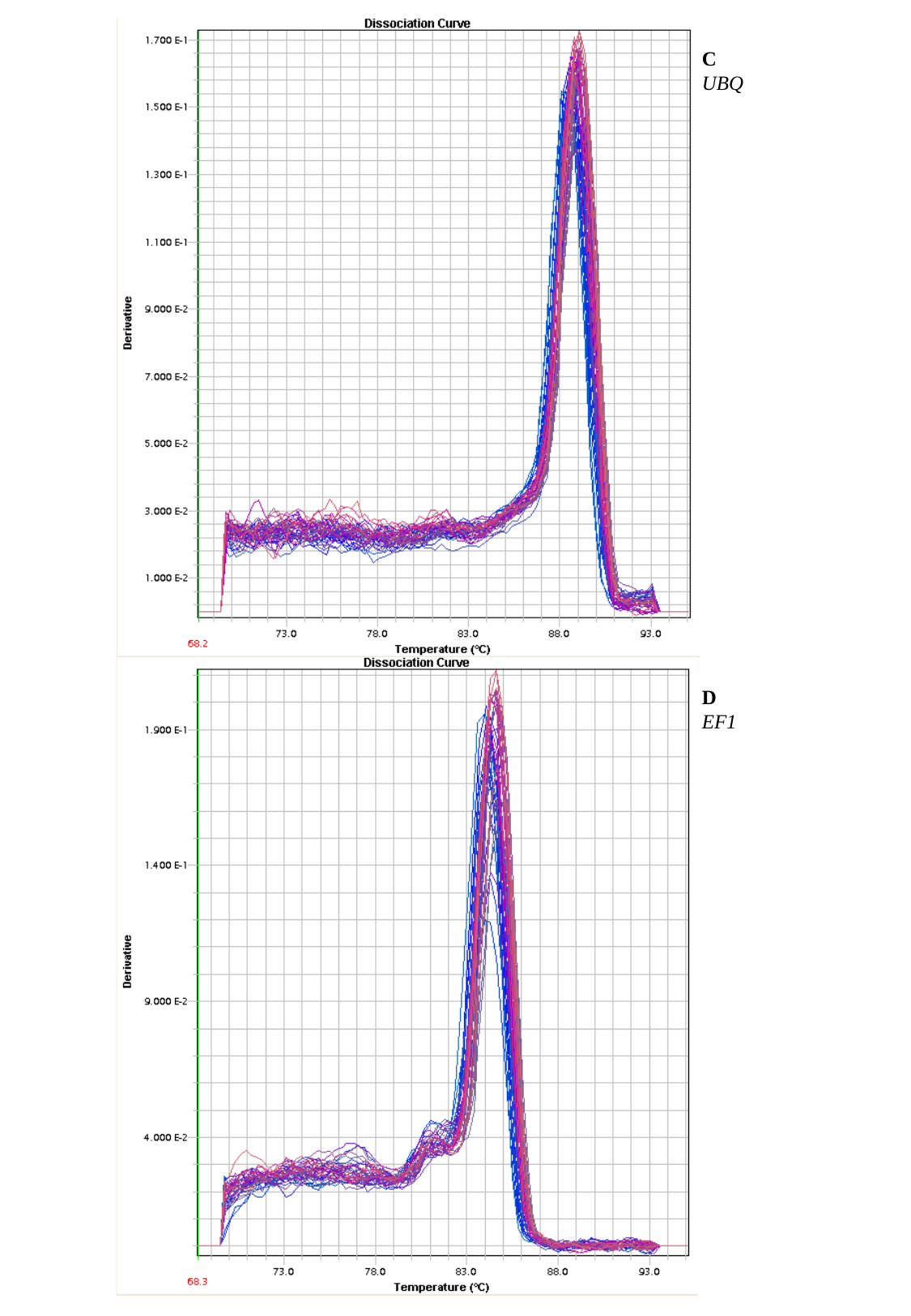

C
UBQ
D
EF1

## Slide 3
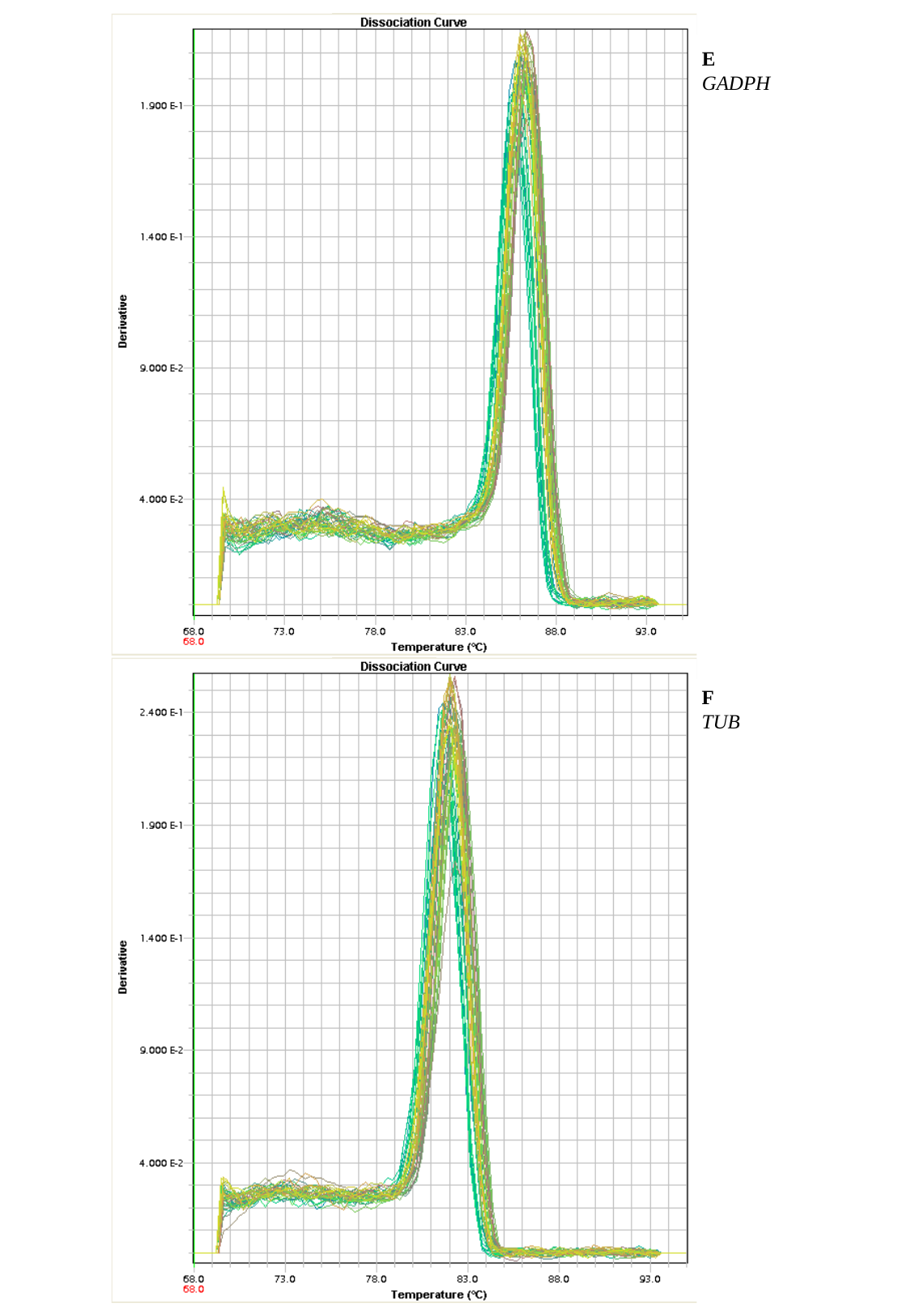

E
GADPH
F
TUB

## Slide 4
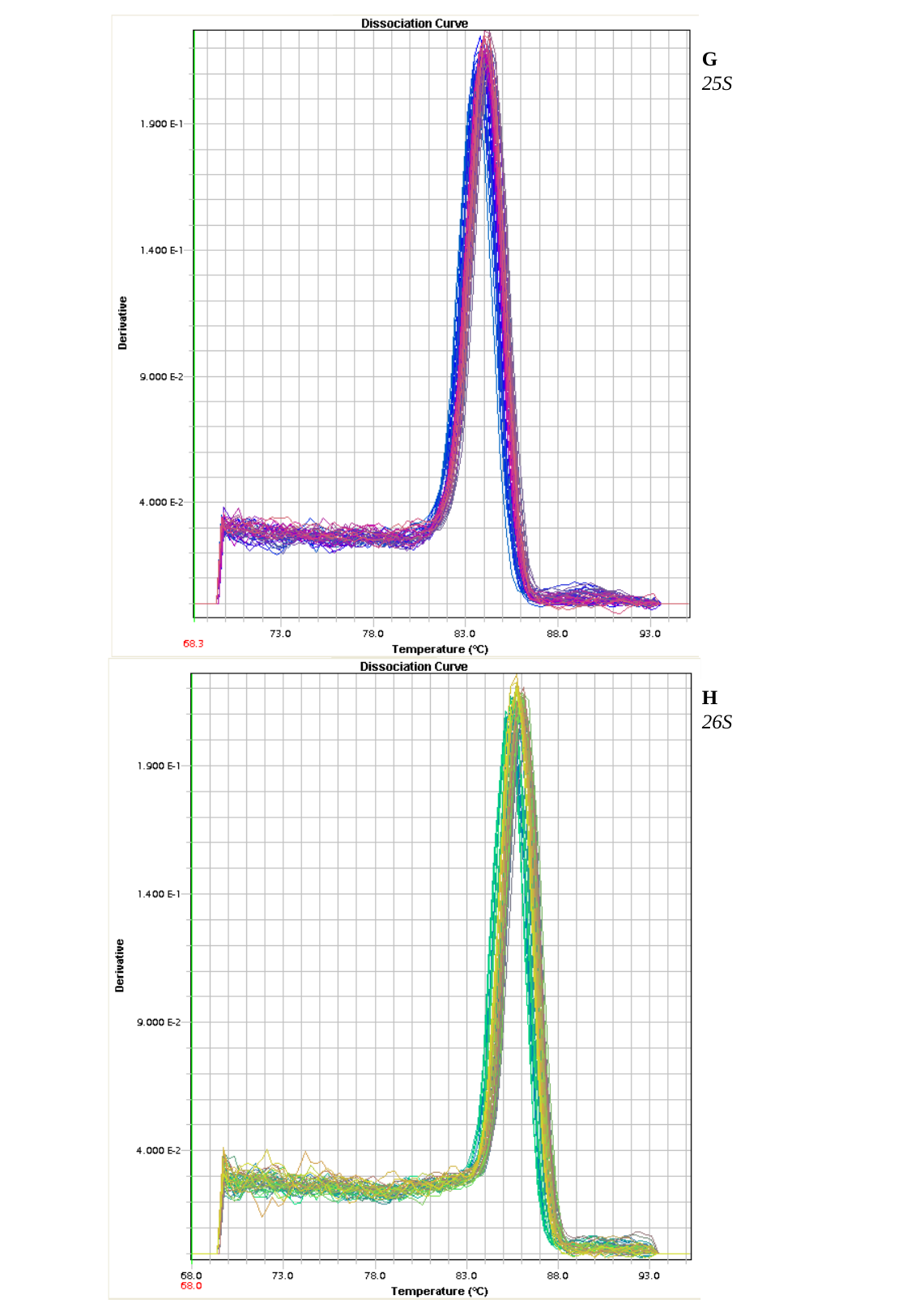

G
25S
H
26S

## Slide 5
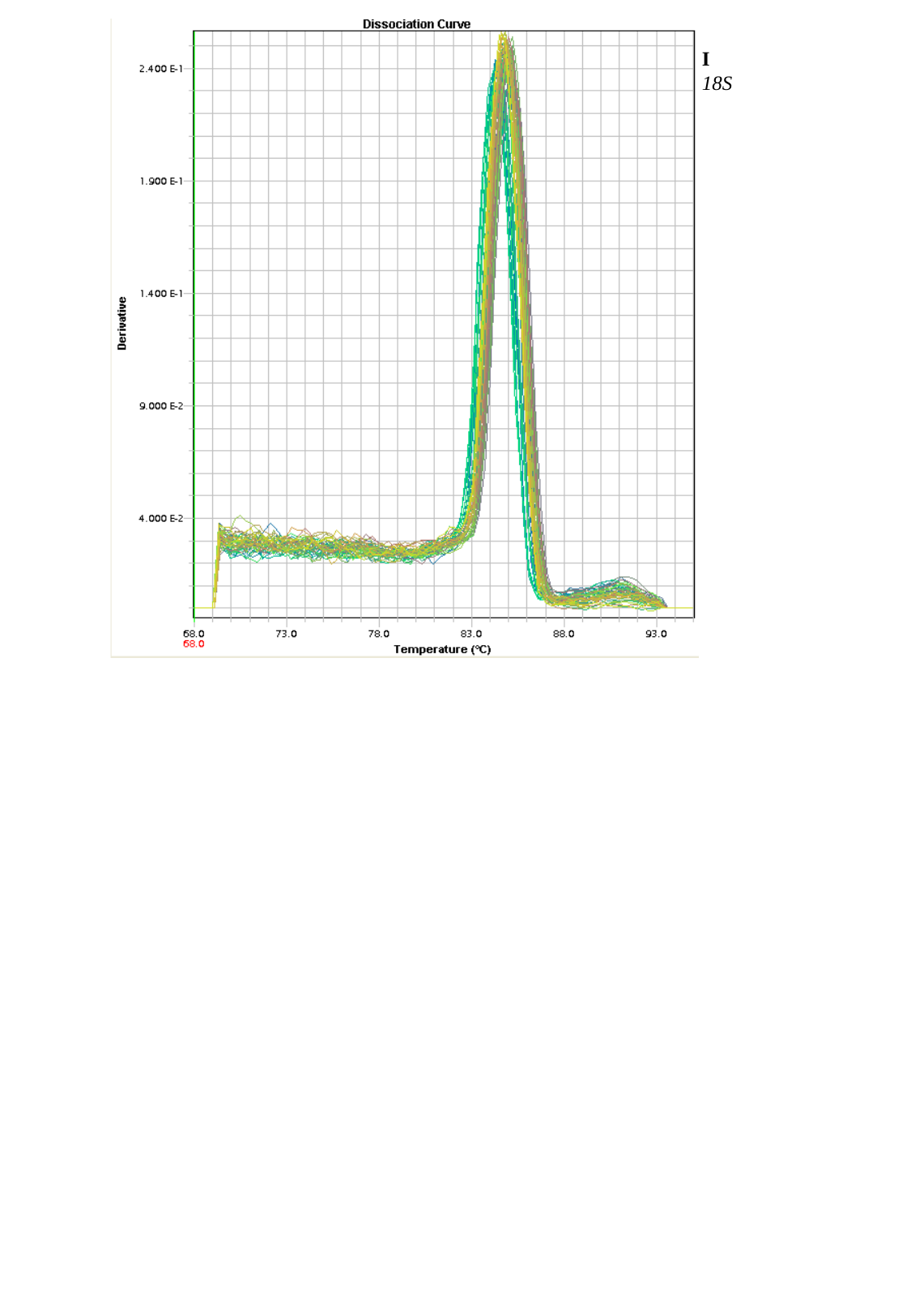

I
18S

## Slide 6
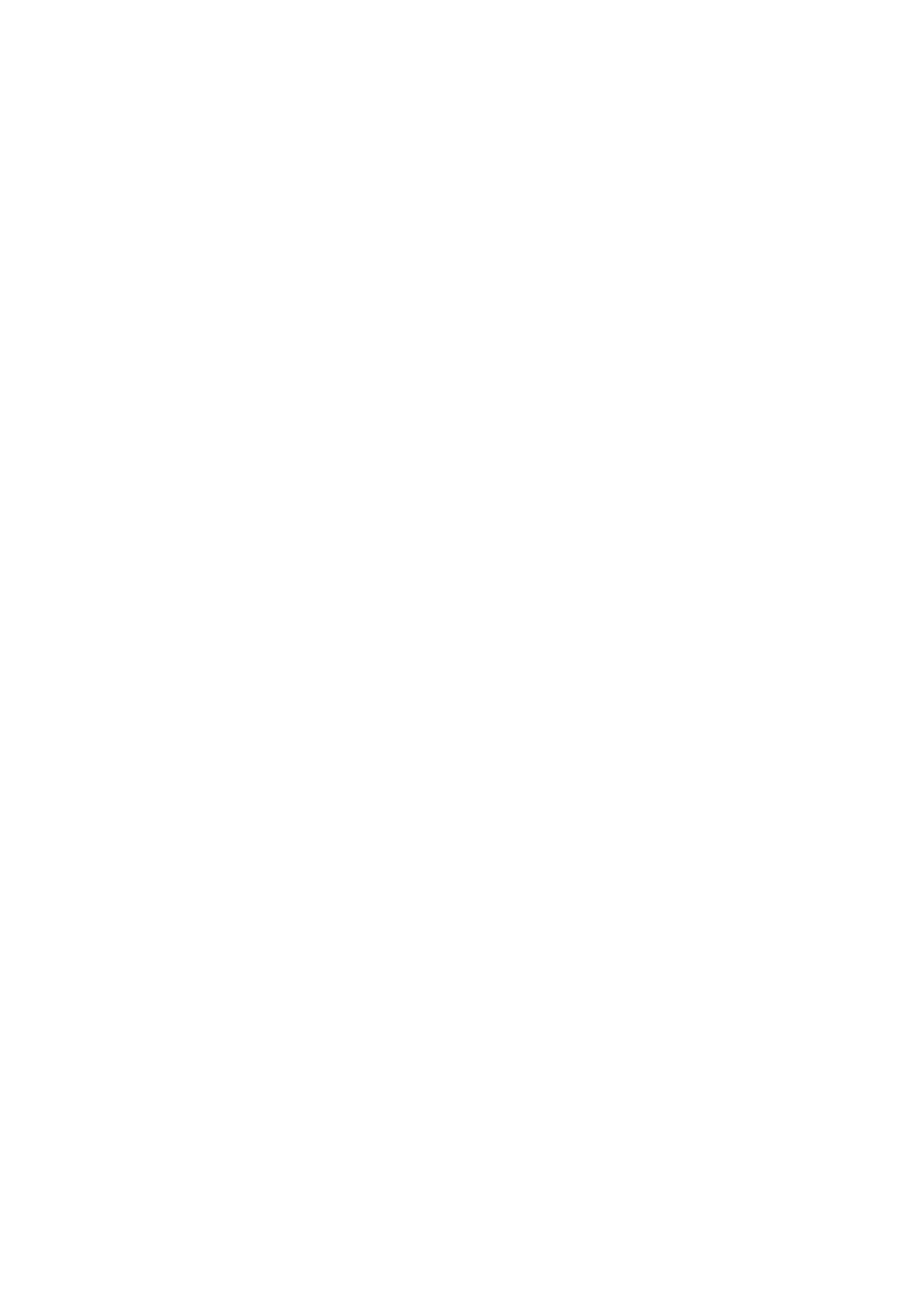

Supplement: Additional file 2 — Figure S1. Primer specificity test. - melting curve generated for RUBISCO. Melting curves generated for RUBISCO (A), ACT (B), UBQ (C), EF1 (D), GAPDH (E), TUB (F), 25S (G), 26S (H) and 18S (I). [file 1756-0500-5-18-S2.PPT]
